# Role of ferroptosis in neuroimmunity and neurodegeneration in multiple sclerosis revealed by multi‐omics data

**DOI:** 10.1111/jcmm.18396

**Published:** 2024-05-27

**Authors:** Tao Wu, Shangwei Ning, Huixue Zhang, Yuze Cao, Xia Li, Junwei Hao, Lihua Wang

**Affiliations:** ^1^ Department of Neurology Xuanwu Hospital, Capital Medical University Beijing China; ^2^ National Center for Neurological Disorders Beijing China; ^3^ College of Bioinformatics Science and Technology Harbin Medical University Harbin China; ^4^ Department of Neurology The Second Affiliated Hospital, Harbin Medical University Harbin China; ^5^ Department of Neurology Peking Union Medical College Hospital, Chinese Academy of Medical Sciences and Peking Union Medical College Beijing China

**Keywords:** ferroptosis, multi‐omics, multiple sclerosis, neurodegeneration, neuroimmunity

## Abstract

Previous studies have found that ferroptosis plays an important role in a variety of neurological diseases. However, the precise role of ferroptosis in the multiple sclerosis patients remains uncertain. We defined and validated a computational metric of ferroptosis levels. The ferroptosis scores were computed using the AUCell method, which reflects the enrichment scores of ferroptosis‐related genes through gene ranking. The reliability of the ferroptosis score was assessed using various methods, involving cells induced to undergo ferroptosis by six different ferroptosis inducers. Through a comprehensive approach integrating snRNA‐seq, spatial transcriptomics, and spatial proteomics data, we explored the role of ferroptosis in multiple sclerosis. Our findings revealed that among seven sampling regions of different white matter lesions, the edges of active lesions exhibited the highest ferroptosis score, which was associated with activation of the phagocyte system. Remyelination lesions exhibit the lowest ferroptosis score. In the cortex, ferroptosis score were elevated in neurons, relevant to a variety of neurodegenerative disease‐related pathways. Spatial transcriptomics demonstrated a significant co‐localization among ferroptosis score, neurodegeneration and microglia, which was verified by spatial proteomics. Furthermore, we established a diagnostic model of multiple sclerosis based on 24 ferroptosis‐related genes in the peripheral blood. Ferroptosis might exhibits a dual role in the context of multiple sclerosis, relevant to both neuroimmunity and neurodegeneration, thereby presenting a promising and novel therapeutic target. Ferroptosis‐related genes in the blood that could potentially serve as diagnostic and prognostic markers for multiple sclerosis.

## 1 INTRODUCTION

Multiple sclerosis (MS) is the most common autoimmune disease of the central nervous system in adults, affecting more than 2.3 million people worldwide.^1^ MS has a complex pathogenesis characterized by the dual manifestations of neuroimmune inflammation and neurodegeneration.^2^ The pathogenesis of MS involves a variety of immune cells, including innate immune cells in the brain (such as microglia) and peripheral infiltrating immune cells (T cells, B cells and monocyte‐derived cells).^3^, ^4^ Peripheral infiltrating immune cells are associated with acute inflammatory infiltration and demyelination in MS, while microglia may be related to neurodegeneration in the later stages of the disease.^3^ However, the precise mechanisms underlying MS pathogenesis remain poorly elucidated.

Ferroptosis is a specific form of cell death biochemically and morphologically distinct from apoptosis and necrosis, characterized by mitochondrial shrinkage, accumulation of lipid peroxides, and regulated in an iron‐dependent manner. In recent years, several studies have revealed the significant role of ferroptosis in Experimental autoimmune encephalomyelitis (EAE) mice, a murine model of MS. Rothammer et al.^5^ discovered that treatment with a G9a inhibitor in EAE mice can restore the expression of anti‐ferroptotic genes, thereby reducing inflammation‐induced neuronal loss. Luoqian et al.^6^ observed an upregulation of the ferroptosis‐related gene *ASCL4* in MS. In EAE mouse model, they identified a potential connection between ferroptosis and T cell activation,^6^ further emphasizing the importance of exploring this biological process in the context of MS. However, ethical considerations and the scarcity of specimens have limited research on ferroptosis in MS patients.

Recent technological advancements in single‐cell/nucleus RNA‐sequencing (sc/snRNA‐seq) and spatial multi‐omics have become crucial tools for investigating complex diseases at both cellular and spatial levels. Prior studies employing snRNA‐seq have revealed oligodendrocyte heterogeneity,^7^ neuronal vulnerability and diversity,^8^ and lymphocyte‐microglia‐astrocytes axis^9^ within MS lesions. Spatial transcriptomics analysis has also identified early degeneration pathways and their spatial distribution in MS.^10^ These advancements in snRNA‐seq and spatial multi‐omics present new avenues for gaining deeper insights into MS. However, there is a scarcity of studies that have utilized snRNA‐seq and spatial multi‐omics data to analyse the ferroptosis in individuals with MS.

Here, we defined a computational metric of ferroptosis levels and identified the ferroptosis landscape in neuroimmunity and neurodegeneration in individuals with MS by integrating snRNA‐seq, spatial transcriptomics, and spatial proteomics data. First, using snRNA‐seq data from white matter lesions, which are often demyelinating lesions infiltrated by immune cells, we elucidate the ferroptosis score in the function of immune cells. Second, employing cortical snRNA‐seq data, we uncover the relevance of ferroptosis score and neurodegeneration during the later stages of MS. Additionally, we analyse the spatial distribution of ferroptosis score using spatial transcriptomics data and validate our findings with spatial proteomics data. Finally, we develop a machine learning model based on ferroptosis‐related genes, facilitating MS diagnosis and prognostication. In summary, this study provides insights into the potential relevance between ferroptosis and white matter neuroimmune inflammation and cortical neurodegeneration in MS, enhancing our understanding of its pathogenesis.

## 2 MATERIALS AND METHODS

### 2.1 Data source

All data in this study are available in Table S1. All data are sourced from publicly available datasets. White matter snRNA‐seq data included a total of 84,222 nuclei from nine MS patients and eight controls.^7^, ^9^ Grey matter snRNA‐seq data included 48,919 nuclei from 19 MS and 16 controls.^8^ Spatial transcriptomics data includes 83,256 spots from 174 slices of 13 MS and 4 controls.^10^ Spatial proteomics data includes high‐sensitivity mass spectrometry data from 13 MS and 7 controls.^10^ Paired cerebrospinal fluid (CSF) and peripheral blood (PB) scRNA‐seq data were from 12 RRMS patients and 3 controls, containing 130,015 cells.^11^ The bulk RNA data of PB used for machine learning includes seven microarray data (1453 samples),^12^, ^13^, ^14^, ^15^, ^16^, ^17^, ^18^ and 2 RNA‐sequencing data for model testing (70 samples).^19^, ^20^ One bulk RNA data with detailed relapse time was used for survival analysis (65 samples).^21^

### 2.2 Sn‐RNA and Sc‐RNA data analysis

The expression matrix is normalized and scaled by the ‘NormalizeData’ and ‘ScaleData’ with standard parameters in the ‘Seurat’ package (https://cran.r‐project.org/web/packages/Seurat/index.html). Principal component analysis (PCA) was performed. Dataset integration and batch effect removal were performed using the Seurat‐compatible Harmony pipeline (‘RunHarmony’ function) of the ‘harmony’ package. Subsequently, the integrated objects were visualized by t‐distributed Stochastic Neighbour Embedding (tSNE) or Uniform Manifold Approximation and Projection (UMAP) depending on the number of cells/nuclei. Cell filtering is performed based on the following parameters^22^: min.cells = 10, min.features = 200. The top 2000 variable expressed genes were determined by the ‘FindVariableFeatures’ function of the ‘Seurat’ package. The ‘FindClusters’ function was used to classify cells into different clusters. Cell types were annotated according to specific cell markers. Immune cell clusters in the brain white matter were annotated based on the enrichment of previously established gene sets for specific cell types.^9^

### 2.3 Calculation of ferroptosis score

Ferroptosis scores were calculated using the AUCell method.^23^ AUCell employs area under the curve (AUC) to calculate whether the input gene set is enriched in the expressed genes of each cell. Since AUCell's scoring method is based on rankings, it is not affected by gene expression units and normalization procedures. Also, due to its individual evaluation of each cell, it is suitable for the application of larger data sets. In detail, ferroptosis scores were calculated for each cell or tissue block by the ‘AUCell_calcAUC’ function of the ‘AUCell’ package, where the ‘aucMaxRank’ parameter was set to 10% of the number of genes detected in the dataset. We downloaded the ferroptosis gene set from Molecular Signatures Database (MSigDB, https://www.gsea‐msigdb.org/gsea/msigdb/human/geneset/WP_FERROPTOSIS.html), and its enrichment score was used as the ferroptosis score, reflecting the activation level of the ferroptosis. The reliability of the ferroptosis score was assessed using cells undergoing ferroptosis induced by six different ferroptosis inducers (Table S2).^24^, ^25^, ^26^, ^27^ We also calculate other enrichment values include ferroptosis drivers, ferroptosis suppressors and ferroptosis markers gene set. Ferroptosis regulatory genes (ferroptosis drivers, ferroptosis suppressors and ferroptosis markers) were downloaded from FerrDb V2.^28^

### 2.4 Cell communication analysis

CellChat is used to quantitatively infer the cell‐to‐cell communication networks.^29^ CellChat objects are created by the R package ‘CellChat’ (https://www.github.com/sqjin/CellChat). The ‘CellChatDB.human’ ligand‐receptor interaction database was used as preference data. Cell communication analysis was performed using the default parameter set. Cell communication networks are aggregated using the ‘aggregateNet’ function. Finally, we visualized the strength of communication between cell clusters and the expression of signalling genes using the ‘netVisual_circle’ and ‘netVisual_bubble’ functions respectively.

### 2.5 Cell trajectory inference

Single cell trajectory was analysed using matrix of cells and gene expressions by Monocle 2.^30^ Cell progression genes were defined based on 3009 expressed genes (DEGs) identified by comparing the transcriptomics among different phagocyte subtypes. By assessing trends in the expression of these genes in each cell, the trajectory of cellular development is revealed. The pseudotime values in the output represent the temporal order of cell development. The standard ‘estimateSizeFactors’ and ‘estimateDispersions’ pipelines were performed to build lineage trajectories and branch points with default parameters. Use the ‘plot_cell_trajectory’ function to visualize cell trajectories, and use ‘plot_genes_in_pseudotime’ to display changes in the relative expression of homeostatic state and active state related genes.

### 2.6 Functional enrichment analysis

All DEGs were obtained from comparisons within the same tissue type. DEGs of cell clusters in snRNA‐seq data were identified by using the ‘FindAllMarkers’ function with ‘logfc.threshold = 0.25’. DEGs in the bulk RNA data were determined using the R package ‘limma’^31^ which implements an empirical Bayesian method. To ensure an adequate number of candidate genes for model construction, the cutoff *p*‐value for DEGs of blood bulk RNA is set at 0.05. Gene annotation enrichment analysis was performed using the ‘clusterProfiler’ R package.^32^ KEGG pathway analysis was performed using ‘enrichKEGG’. Gene Ontology (GO) enrichment analysis was performed using ‘enrichGO’, ont = ‘BP’. The construction and visualization of GO network graphs were performed using ‘Enrichplot’ (https://github.com/GuangchuangYu/enrichplot). We also determined the change of ferroptosis in spatial transcriptomics tissue blocks between MS patients and controls by ‘GSEA’ function.^33^

### 2.7 Spatial multi‐omics data processing

Use the ‘CreateSeuratObject’ function in ‘Seurat’ to create a spatial transcriptomics analysis object. Use ‘Read10X_Image’ to integrate H&E images. Count matrices were normalized with ‘SCTransform’ and analysed using a pipeline with standard parameters. SnRNA‐seq data were integrated to perform cell type deconvolution of each spot in the spatial transcriptomics. The data for integration were human cortical snRNA‐seq data from five different sources, including reference data from the Allen brain atlas and scRNA‐seq data from blood immune cells.^7^, ^8^ Data integration was performed using enrichment score‐based deconvolution implemented with AUCell.^23^ Based on the previously established cell type genes that integrated snRNA‐seq data,^10^ we calculated the cell type score for each spot using ‘AUCell_calcAUC’. ‘SpatialFeaturePlot’ was used to map ferroptosis score, ferroptosis‐related scores, and cell type scores.

We performed quality control on the proteomics data to filter out quantifiable proteins in less than 30% of the samples. GS, defined as the correlation (absolute value) between the gene and the trait, is used to quantify the association of individual genes with our trait of interest (weight).^34^ ‘WGCNA’ package^35^ was used to calculate GS of every overlapping gene in spatial transcriptomics and spatial proteomics respectively, and the correlation analysis was performed.

### 2.8 Construction of diagnosis model based on machine learning

The classic machine learning package ‘e1071’ was used for machine learning (https://cran.r‐project.org/web/packages/e1071/index.html). The matrix‐based visualization showing intersections of DEGs were through UpSetR.^36^ We extracted 24 ferroptosis‐related genes with the most evidence in all bulk RNA data sets. For the comparability between data sets, the extracted data were standardized by Z‐score according to the mean and standard deviation (missing values were filled with the mean value). The ‘svm’ function was used to build the model, kernel = ‘linear’. Use the ‘predict’ function to predict the model on the validation and test sets. The final output of the model is defined as the decision values. The diagnostic performance of the model was evaluated according to the AUC on the basis of the ‘pROC’ (https://cran.r‐project.org/web/packages/pROC) R package.

### 2.9 Statistical analysis

All statistical analyses and visualizations were performed using R 4.2.2 software. Spearman's correlation analysis was used to clarify the relationship between two continuous variables. The Wilcoxon rank‐sum test or *t*‐test was used to compare differences in continuous variables between groups. A *p*‐value below 0.05 was considered statistically significant.

## 3 RESULTS

### 3.1 Higher ferroptosis scores are observed at the edges of active lesions in white matter

Due to the lack of specific markers for ferroptosis, we attempted to quantify the level of ferroptosis by defining the ferroptosis score as the enrichment level of genes associated with ferroptosis. Subsequently, we utilized three supplementary methodologies to evaluate the reliability of the ferroptosis score in representing the state of ferroptosis. Initially, we assessed whether the ferroptosis score was elevated in ferroptotic cells compared to normal cells. Comparing six different inducers of ferroptosis across various cell lines, we consistently observed a rise in the ferroptosis score in all cells undergoing induced ferroptosis (average increase of 13.7%, Table S2). Moreover, based on gene expression‐based enrichment analysis, we found significant positive correlations between the ferroptosis score and pathways involving oxidative phosphorylation, redox homeostasis, reduced mitochondrial activity, and altered mitochondrial morphology, aligning with typical biochemical changes in ferroptosis (Figure S1A). Additionally, we discovered that the ferroptosis score effectively distinguished known normal cells from ferroptotic cells in five independent datasets (comprising 39 cell line samples induced by six different ferroptosis inducers), with the area under the curve (AUC) surpassing that of the ferroptosis marker gene set (Table S2). In summary, these results demonstrate a significant association of the ferroptosis score with key ferroptotic functionalities, exhibiting superior performance compared to a mere set of ferroptosis marker genes.

**FIGURE 1:**
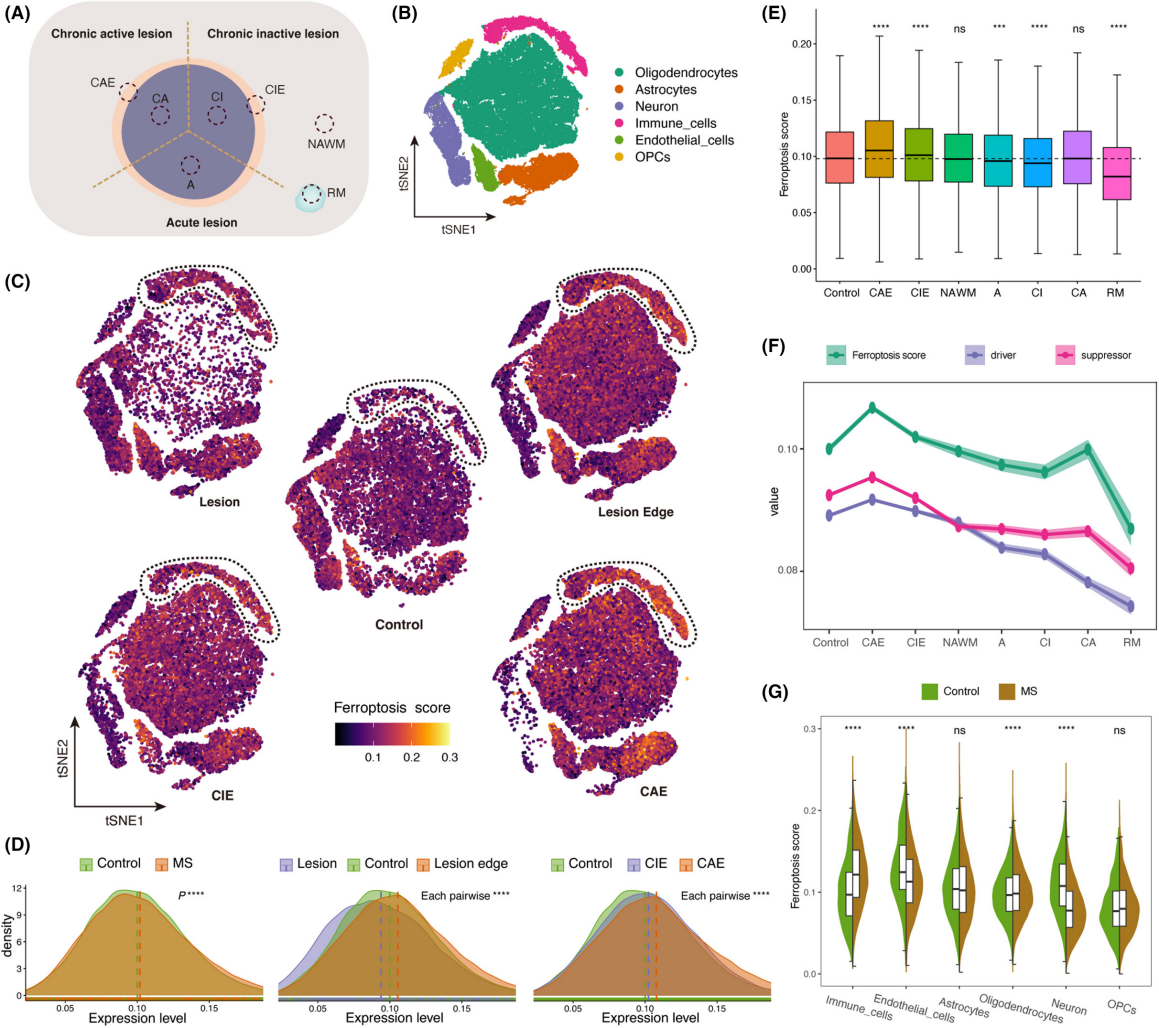
SnRNA‐seq data reveals ferroptosis scores in white matter lesions of MS. (A) Schematic diagram of the seven sampling regions in MS brain tissues. (B) The tSNE projections of cell clusters (*n* = 84,222 nuclei from eight controls and nine MS patients). (C) The tSNE projection of ferroptosis score on control (middle), MS lesions (upper left, including acute lesion, CI and CI), MS lesion edges (upper right, including CIE and CAE), CIE (lower left) and CAE (lower right). Brightness represents ferroptosis score. Immune cells are circled. (D) Density plot of ferroptosis score distribution in different groups: control versus MS (left), control, MS lesions, and MS lesion edges (middle), control, CIE, and CAE (right). Dashed lines represent means, the *t*‐test was employed. (E) Comparison of ferroptosis score between seven sampling regions and the control (the dashed line represents the median ferroptosis score of the control, the Wilcoxon rank‐sum test was employed). (F) Line graph of the ferroptosis score (green), and enrichment scores of ferroptosis driver (lavender), and ferroptosis suppressor (red) at different sampling regions. Points represents the means, and the width of the ribbon represents the 95% confidence interval (CI). (G) Faceted violin plot comparing ferroptosis scores in control (green) and MS (brown) in different cell types. Compared with the control, the *t*‐test was employed. Boxplot centred on median, bounds defined between the 25th and 75th percentile. *p < 0.05, **p < 0.01, ***p < 0.001. A, acute lesions; CA, chronic active lesion; CAE, chronic active lesion edge; CI, chronic inactive lesion; CIE, chronic inactive lesion edge; MS, multiple sclerosis; NAWM, normal appearing white matter; OPCs, oligodendrocyte progenitor cells; RM, remyelinated lesions; tSNE, t‐distributed Stochastic Neighbour Embedding.

We integrated two available snRNA‐seq datasets of white matter lesions,^7^, ^9^ encompassing multiple sampling regions: chronic active lesions (CA), chronic inactive lesions (CI), chronic active lesion edge (CAE), chronic inactive lesion edge (CIE), acute lesions (A), normal appearing white matter (NAWM), remyelination lesions (RM), and control white matter (Figure 1A). We performed dimensionality reduction and cell clustering on the integrated data to identify distinct cell clusters (Figure 1B, Figure S1B).

As described in method, we calculated the ferroptosis score based on ferroptosis gene set downloaded from MSigDB. To comprehensively display the changes in ferroptosis‐related genes, we calculated the enrichment values of ferroptosis markers, drivers, and suppressors downloaded from the FerroDB V2 database.^28^ We compared the ferroptosis score across various MS lesions and control white matter (Figure 1C,D). Comparative analysis of ferroptosis score between total MS white matter and control white matter revealed an elevation in the ferroptosis score of MS lesions compared to control white matter (Figure 1D). Furthermore, upon comparing the ferroptosis score between the lesions and the edges of lesions, we discovered that the ferroptosis score were lower in the lesions compared to the control, whereas the ferroptosis score at the lesion edges were significantly higher than those of the control (Figure 1D). This observation may indicate the activation of ferroptosis at the lesion edge, suggesting a redistribution of iron in MS lesions, specifically the accumulation of iron from the lesion's interior to its periphery.

We compared the ferroptosis score between the edges of active lesions and the edges of inactive lesions, revealing that the CAE exhibited higher ferroptosis score than the CIE (Figure 1C,D). Additionally, we compared the ferroptosis score among other regions, including acute lesions, RM, NAWM and control white matter. Among all regions, the CAE consistently displayed the highest ferroptosis score, while RM exhibited the lowest ferroptosis score (Figure 1E). Furthermore, we evaluated the enrichment of ferroptosis drivers and ferroptosis suppressors. The results consistently indicated that the CAE had the highest value (Figure 1F). Interestingly, the enrichment of ferroptosis drivers and ferroptosis suppressors exhibited similar trends, implying the existence of a coordinated regulatory mechanism in MS in response to ferroptosis.

Additionally, we examined variations in ferroptosis score among different cell types. In comparison to controls, immune cells exhibited the most significant increase in ferroptosis score within MS white matter, whereas neurons and endothelial cells displayed decreased ferroptosis score (Figure 1C,G, Figure S1C). This finding suggests that immune cells, including microglia, peripheral infiltrating monocytes, lymphocytes, etc., play a crucial role in driving the changes observed in ferroptosis score within white matter lesions of MS patients. Notably, among these immune cell subtypes, phagocytes (microglia and monocyte‐macrophages) are the primary cells with the capability to phagocytose iron in the brain.

### 3.2 Elevated ferroptosis scores are associated with phagocytic activation in white matter lesions

**FIGURE 2:**
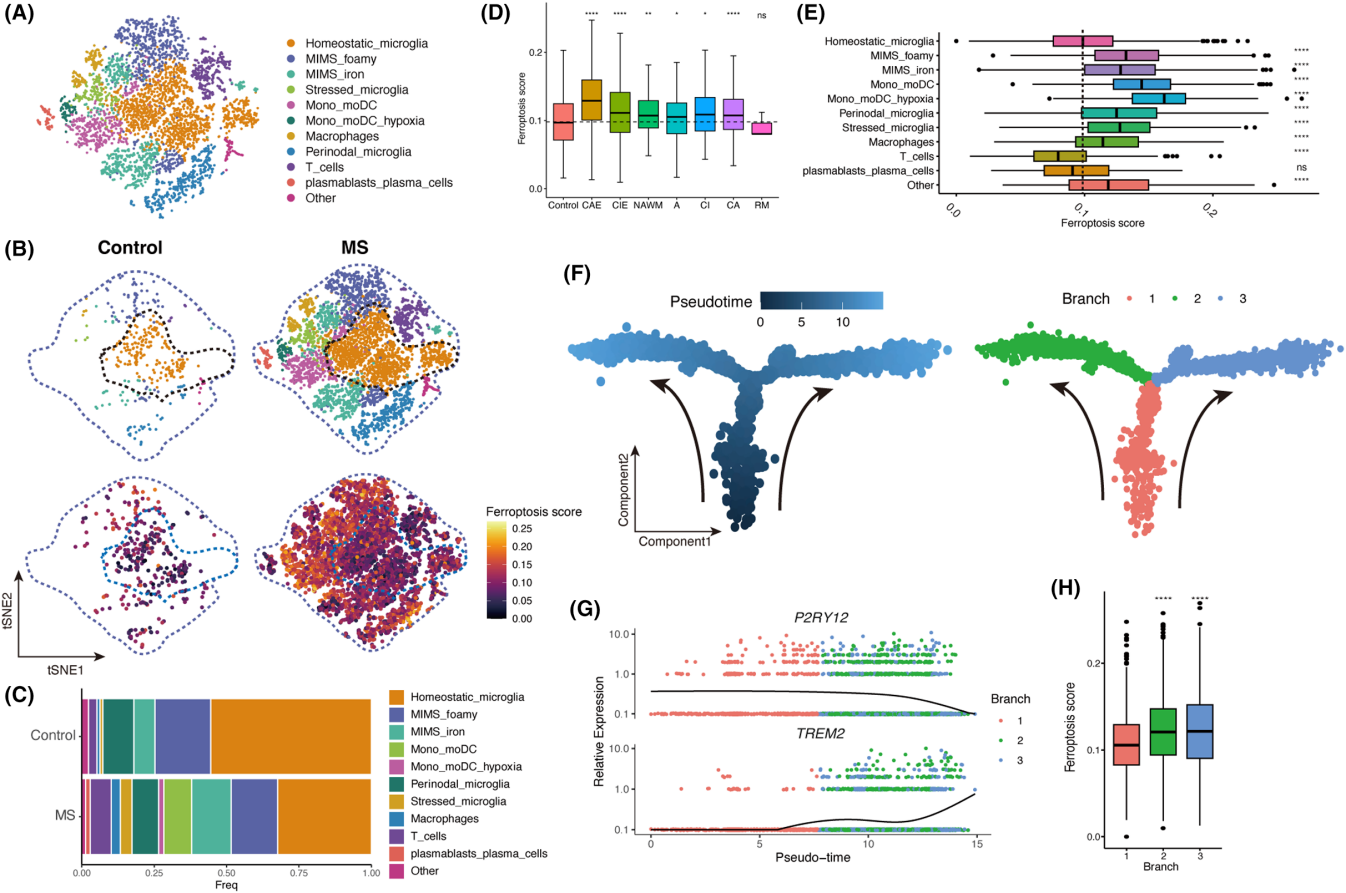
Higher ferroptosis scores in MS white matter lesions are associated with phagocyte activation. (A) tSNE projections of immune cell clusters (*n* = 7048 nuclei). (B) Immune cell distribution between controls and MS patients, and the projection plot of ferroptosis score on tSNE. (C) Stacked column chart of the distribution of immune cell clusters in control and MS. (D) Comparison of the ferroptosis scores of immune cells at different sampling regions (the dashed line represents the median ferroptosis score of the control, the Wilcoxon rank‐sum test was employed). (E) The comparison of ferroptosis scores among different immune cell clusters (dashed line represents median ferroptosis score of homeostatic microglia). (F) Pseudotime trajectory of phagocytes (left). The locations of the three pseudotime branches on the trajectory (right). Arrows represent pseudotime directions. (G) Changes of phagocytic homeostatic‐state genes (*P2RY12*) and active‐state genes (*TREM2*) with pseudotime. (H) Comparison of ferroptosis scores in three pseudotime branches (compared with branch 1, the Wilcoxon rank‐sum test was employed). *p < 0.05, **p < 0.01, ***p < 0.001. A, acute lesions; CA, chronic active lesion; CAE, chronic active lesion edge; CI, chronic inactive lesion; CIE, chronic inactive lesion edge; MIMS, microglia inflamed in MS; mono/moDC, monocytes/dendritic cells; NAWM, normal appearing white matter; RM, remyelinated lesions; tSNE, t‐distributed Stochastic Neighbour Embedding.

Considering the significant increased ferroptosis score in immune cells within MS white matter lesions, our analysis focused specifically on immune cells. Utilizing a previously established immune cell clustering method for MS white matter,^9^ we categorized immune cells into distinct subtypes including microglia (resident phagocytes), monocyte‐derived cells (peripherally infiltrating phagocytes), B cells, T cells and others (Figure 2A, Figure S2A,B). We compared the distribution of immune cells between MS and control white matter and determined the ferroptosis scores for each cell type (Figure 2B). As an immune‐inflammatory disease, MS lesions typically exhibit abundant infiltration of immune cells, hence the notable increase in immune cells in MS brain tissue compared to normal brain tissue as depicted in the figure. The results showed a complex and diverse immune cell composition in MS, whereas homeostatic microglia dominated the control white matter (Figure 2C).

We compared the ferroptosis score of immune cells across different MS lesions, and the findings demonstrated higher ferroptosis score in immune cells from MS compared to control. Notably, immune cells in CAE exhibited the highest ferroptosis score (Figure 2D). Upon mapping the ferroptosis score to the cell clusters, we observed that homeostatic microglia, located centrally on the map, displayed relatively low ferroptosis score, whereas activated microglia and monocyte‐derived cells, positioned peripherally, exhibited relatively high ferroptosis score (Figure 2B). Further analysis of individual immune cell clusters revealed significantly higher ferroptosis score in activated microglia (microglia inflamed in MS (MIMS) foamy, MIMS iron, and stressed microglia) and monocyte‐derived cells, particularly in monocytes/dendritic hypoxia cells (mono/moDC hypoxia) with the highest ferroptosis score (Figure 2E). Conversely, T cells and B cells exhibited relatively low ferroptosis score, suggesting that ferroptosis occurrence in white matter lesions may primarily involve phagocyte system activation rather than lymphocytes. This observation aligns with our understanding that the breakdown of myelin sheaths releases substantial amounts of iron, which are predominantly phagocytosed by brain phagocytes.

To validate this hypothesis, we conducted cell development trajectory inference on the brain's phagocyte system. The trajectory originated from branch 1 and bifurcated into branch 2 and branch 3, characterized by MIMS foamy and MIMS iron, respectively (Figure 2F, Figure S2C,D). With increasing pseudotime, the expression of the *P2RY12* gene, indicative of homeostasis, gradually decreased, while the expression of the *TREM2* gene, indicative of activation, progressively increased, reflecting the phagocyte system's activation process (Figure 2G). Subsequently, we compared the ferroptosis score between branch 2 and branch 3 with those of branch 1, revealing significantly higher ferroptosis score in branch 2 and branch 3 (Figure 2H). These findings further confirm the close association between ferroptosis score and phagocyte system activation in MS white matter lesions.

### 3.3 Phagocytes with elevated ferroptosis scores may contribute to neuroimmune inflammation

**FIGURE 3:**
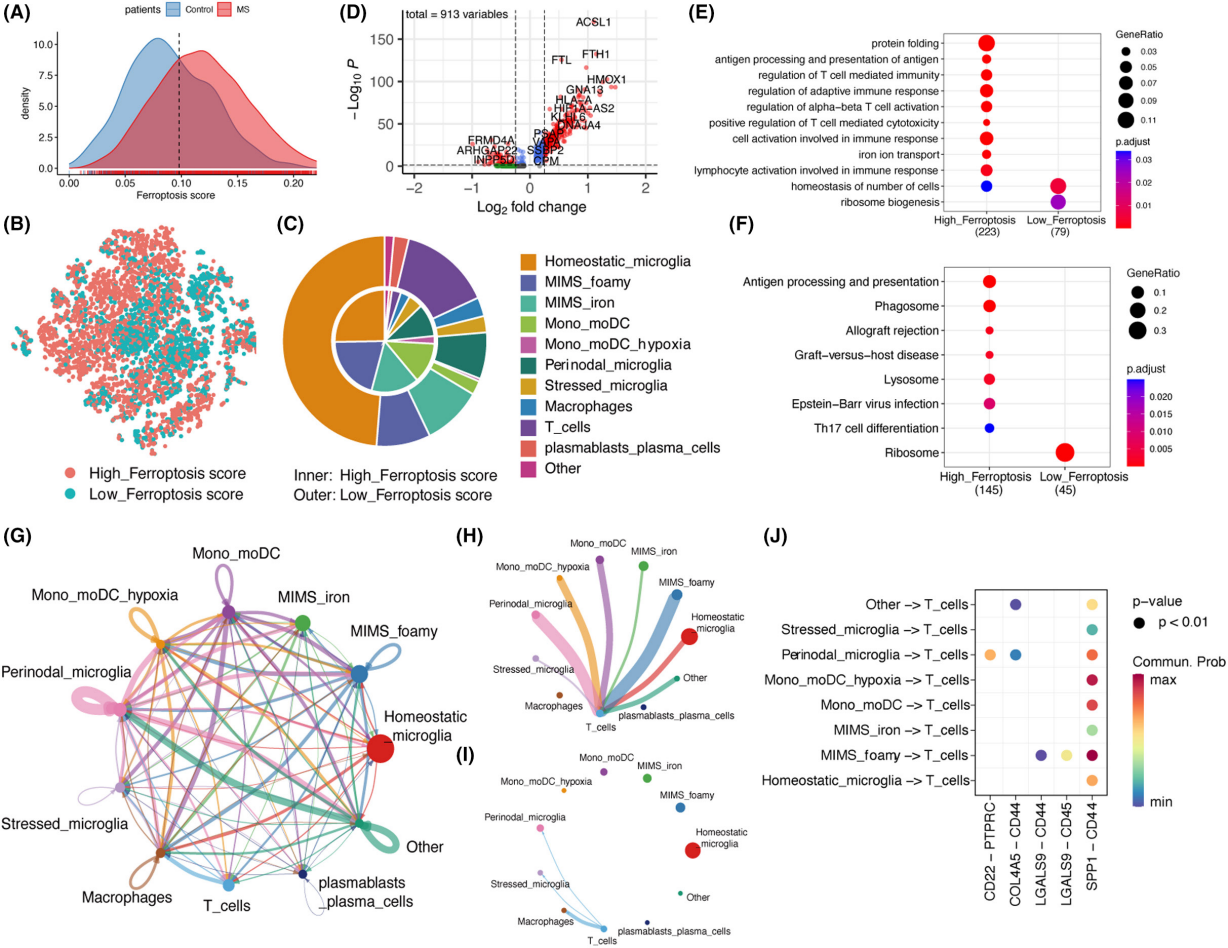
Phagocytes with higher ferroptosis scores may contribute to neuroimmune inflammation. (A) Density plot of the ferroptosis scores of immune cells in controls and MS patients. (B) The tSNE projection of immune cells in high‐ferroptosis score group and low‐ferroptosis score group. (C) Pie chart of the distribution of immune cells in the high‐ferroptosis score group and the low‐ferroptosis score group. (D) Volcano plot of the DEGs of phagocytes in the high‐ferroptosis score group compared to the low‐ferroptosis score group. (E) GO enrichment analysis of DEGs in phagocytes of high‐ferroptosis score group and low‐ferroptosis score group. (F) KEGG functional enrichment analysis of DEGs in phagocytes of high‐ferroptosis score group and low‐ferroptosis score group. (G) Diagram of the cell communication network between immune cell clusters. Circle sizes are proportional to the number of cells in each cell cluster. Edge colours represent cell clusters that signalled and edge width represents the communication strength. (H) Cell communication map of T cells as signal recipients. (I) Cell communication map of T cells as signal senders. (J) Dot plot showing the receptor‐ligand pairs that immune cell clusters communicate with T cells. Dot colour reflects communication probabilities and dot size represents computed *p*‐values. Empty space means the communication probability is zero. *p < 0.05, **p < 0.01, ***p < 0.001. DEGs, differentially expressed genes; GO, Gene Ontology; KEGG, Kyoto Encyclopedia of Genes and Genomes; MIMS, microglia inflamed in MS; mono/moDC, monocytes/dendritic cells; tSNE, t‐distributed Stochastic Neighbour Embedding.

We categorized all immune cells into high‐ferroptosis score and low‐ferroptosis score groups based on the intersection of ferroptosis score between MS and control (Figure 3A). Notably, the low‐ferroptosis score group was predominantly composed of homeostatic microglia, while the high‐ferroptosis score group exhibited a higher proportion of activated cells (Figure 3B,C). Subsequently, we performed differential expression analysis on the phagocytes in both groups, revealing numerous genes with differential expression in the high‐ferroptosis score group (Figure 3D). Further enrichment analysis using KEGG and GO demonstrated that phagocytes in the high‐ferroptosis score group were significantly enriched in T cell activation‐related pathways and antigen presentation pathways (Figure 3E,F, Tables S3–S5).

Additionally, we observed a relatively higher expression of genes encoding major histocompatibility complex class I and II (MHC‐I/II) in phagocytes of the high‐ferroptosis score group, indicating an enhanced antigen presentation capability (Figure S2E). Furthermore, the expression levels of *IL1B* (encoding interleukin‐1β), *PD‐L1* (also known as *CD274*), and *PD‐L2* (also known as *PDCD1LG2*) in phagocytes of the high‐ferroptosis score group were comparatively elevated (Figure S2F). These findings suggest that the ferroptosis score in phagocytes within white matter lesions may be associated with multiple pathways, including T cell activation, antigen presentation, interleukin‐1β secretion and immune checkpoint regulation.

Cell communication analysis reveals extensive intercellular communication among immune cells in white matter (Figure 3G). To explore the communication between the phagocytic system and T cells, we demonstrate the signals received and sent by T cells separately (Figure 3H,I). It is evident that T cells primarily receive regulatory signals from the phagocytic system, rather than reciprocally regulating it. Furthermore, we observed that activated cells exhibited elevated ferroptosis score, such as MIMS‐foamy, mono/moDC and mono/moDC hypoxia cells, exert a stronger regulatory influence on T cells compared to homeostatic microglia. The shared ligand–receptor pair for T cell communication across all phagocyte types is SPP1‐CD44 (Figure 3J). Notably, *CD44* is also a regulatory gene involved in ferroptosis,^37^ making it a potential therapeutic target for simultaneously modulating ferroptosis and T cell activation.

### 3.4 Ferroptosis scores are elevated in neurons in the cortex

**FIGURE 4:**
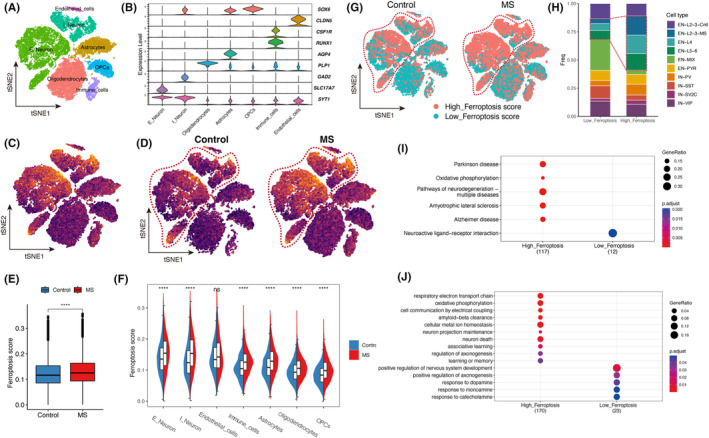
Cortical snRNA‐seq data revealed the relationship between ferroptosis score and neurodegeneration. (A) tSNE projections of cortical cell clusters (*n* = 48,919 nuclei from 16 controls and 19 MS patients). (B) Violin plot of marker genes expression in different cell clusters. (C) Projection of ferroptosis scores on tSNE. (D) Comparison of the control (left) and MS (right) ferroptosis scores on tSNE projection maps. Inside the red circle are neurons. (E) Comparison of ferroptosis scores in control cortex and MS cortex, the *t*‐test was employed. (F) Faceted violin plot comparing ferroptosis scores between control (blue) and MS (red) in different cell clusters, the *t*‐test was employed. (G) tSNE projection map showing the distribution of the high‐ferroptosis score group and the low‐ferroptosis score group in cortical cell clusters. (H) Stacked column chart of the distribution of cortical cell clusters in the high‐ferroptosis score group and the low‐ferroptosis score group. (I) KEGG functional enrichment analysis of DEGs between high‐ferroptosis score group neurons and low‐ferroptosis score group neurons. (J) GO functional enrichment analysis of DEGs between high‐ferroptosis score group neurons and low‐ferroptosis score group neurons. *p < 0.05, **p < 0.01, ***p < 0.001. tSNE, t‐distributed Stochastic Neighbour Embedding.

With disease progression, MS manifests neurodegenerative characteristics, characterized by a certain degree of cortical grey matter atrophy and functional decline. To investigate the involvement of ferroptosis in the neurodegenerative processes of MS patients, we examined snRNA‐seq data^8^ obtained from the cortical region of MS patients. Subsequently, we employed cell clustering techniques to categorize the cortical cells into distinct clusters (Figure 4A,B, Figure S3A–C).

We calculated the ferroptosis score for each cell using the same method as described above (Figure 4C). Consistent with the findings in the MS white matter, we observed a significant elevation in ferroptosis score within the MS cortex compared to the control group (Figure 4D,E). Furthermore, we conducted a separate analysis of ferroptosis score variations for each cell type. Unlike the neurons in the MS white matter, neurons in the MS cortex exhibited significantly higher ferroptosis score compared to the control group, encompassing both excitatory and inhibitory neurons (Figure 4F). This observation might suggest the activation of ferroptosis in MS cortical neurons.

Using the similar method mentioned above, we classified all cells in the cortex into high‐ferroptosis score and low‐ferroptosis score groups (Figure 3D). Analysis revealed a higher presence of high‐ferroptosis score neurons in the MS cortex compared to the control cortex (Figure 4G). Additionally, we examined the distribution of neuron types within the high‐ferroptosis score and low‐ferroptosis score groups. The results demonstrated that the high‐ferroptosis score group had a greater proportion of excitatory neurons in the L2‐3‐MS, L4, and L5‐6 layers compared to the low‐ferroptosis score group (Figure 4H). These findings suggest that excitatory neurons in these specific layers may exhibit increased vulnerability to ferroptosis, which might be a potential explanation for the manifestation of slowed responses and fatigue in MS patients.

### 3.5 Ferroptosis score is associated with degenerative functional changes of neurons

To investigate the association between ferroptosis score and neuronal function, we conducted differential expression and functional enrichment analyses on neurons in high‐ferroptosis score group and low‐ferroptosis score group. KEGG enrichment analysis revealed a significant enrichment of neurodegenerative disease pathways, such as AD, PD, ALS, etc., in neurons exhibiting high ferroptosis score (Figure 4I). These findings suggest that ferroptosis in neurons of MS patients may activate pathways associated with neurodegeneration. GO enrichment analysis revealed that neurons with high ferroptosis score were associated with perturbed pathways related to respiratory electron chain transfer, oxidative phosphorylation, metal ion homeostasis, neuron death, axonogenesis and more (Figure 4J, Tables S6–S8). Notably, neurons with high ferroptosis score showed impaired clearance of Aβ, a process primarily associated with AD pathology, suggesting that this mechanism may contribute to cognitive impairment observed in MS patients. These findings indicate that the expression changes of ferroptosis‐related genes might be associated with neurodegenerative process by involving multiple biochemical and metabolic pathways.

### 3.6 Co‐localization of ferroptosis score and neurodegeneration in spatial transcriptomics analyses

**FIGURE 5:**
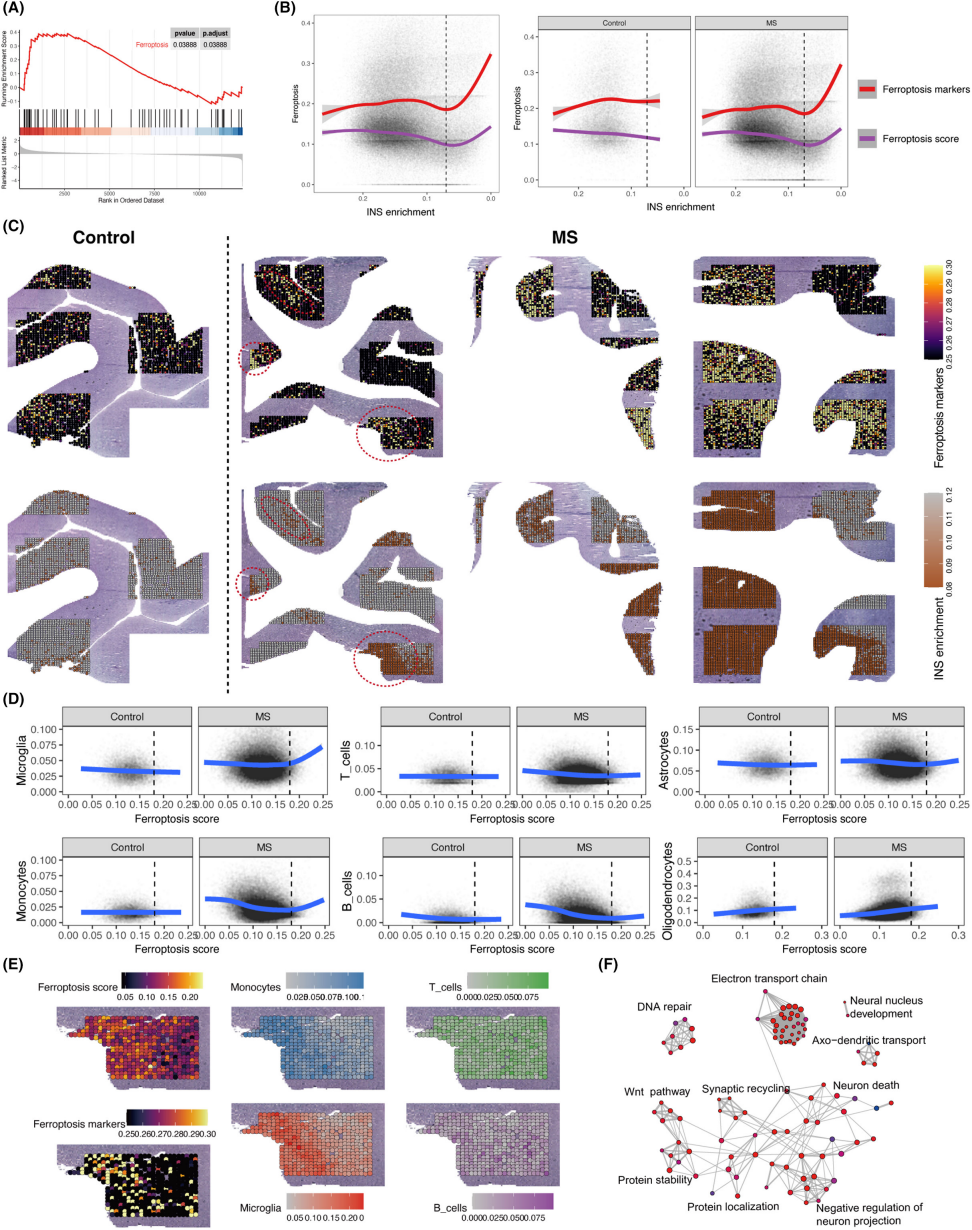
The spatial colocalization of ferroptosis score and neurodegeneration in the spatial transcriptomics. (A) GSEA of ferroptosis pathway in MS patient compared to control samples. (B) The fitting curves of ferroptosis markers and ferroptosis scores with intact neuronal signature enrichment (each point represents a sampling spot, a total of 83,256 spots). Note that the x‐axis is reversed. The lower the intact neuronal signature enrichment score, the more severe the neurodegeneration. The left shows the overall analysis, and the right panel shows the control and MS patients, respectively. (C) Mapping of ferroptosis markers onto grey matter slides (up). Mapping of levels of neurodegeneration (intact neuronal signature enrichment) onto grey matter slides (down). Representative regions are circled by red circles. (D) Changes of the components of cell types with the ferroptosis score, displayed separately for controls and MS patients (each point represents a sampling spot). (E) Projection of immune cells, ferroptosis scores, and intact neuronal signature enrichment onto grey matter slides. (F) GO enrichment modules of the DEGs between high‐ferroptosis score tissue blocks and low‐ferroptosis score tissue blocks (Visualized the top 100 items). DEGs, differentially expressed genes; GM, grey matter; GO, Gene Ontology; GSEA, gene set enrichment analysis.

To comprehensively investigate the spatial distribution of ferroptosis score and neurodegeneration, we utilized cortical spatial transcriptomics data from MS patients.^10^ The dataset comprised 174 tissue blocks obtained from 13 MS patients and four controls, encompassing a total of 83,256 spatial spots. By conducting gene set enrichment analysis (GSEA) on the tissue blocks obtained from MS cortex and control cortex, we observed a significant increased ferroptosis score in the MS cortex compared to the control cortex (Figure 5A). Furthermore, we mapped the changes in ferroptosis‐related genes observed in MS patients onto the ferroptosis pathway map. It was evident that the expression of numerous ferroptosis‐related genes was upregulated in MS patients, while the expression of ferroptosis suppressor genes *GPX4* and *TF* was downregulated (Figure S4A).

Specifically, to elucidate the dynamic process of ferroptosis score, we calculated ferroptosis score for each of the 83,256 spots. The process of neurodegeneration was represented using intact neuronal signature (INS) enrichment, which has been well established and validated.^10^ We found that when the INS enrichment was very low (indicating severe neurodegeneration), there was a consistent increase in ferroptosis score and ferroptosis markers as the INS enrichment score decreased. This observation might suggest that ferroptosis escalate alongside the progression of neurodegeneration. Importantly, this trend was observed exclusively in MS patients and not in controls (Figure 5B), suggesting a distinct role for ferroptosis in neurodegenerative diseases and normal aging. Moreover, we projected the levels of neurodegeneration and the enrichment of ferroptosis markers onto tissue slides to examine their spatial distribution characteristics, revealing a pronounced co‐localization between neurodegeneration and ferroptosis markers (Figure 5C).

We further investigated the impact of cortical ferroptosis score on immune cells. By employing deconvolution, we calculated the composition of different cell types in each spot. The results revealed a significant increase in the abundance of microglia and monocytes in MS cortical tissue when the ferroptosis score surpassed a certain threshold, which contrasted the control samples (Figure 5D). However, such a trend was not observed in T cells and B cells. Furthermore, we observed the co‐localization of microglia, monocytes, and ferroptosis score, while B cells and T cells did not exhibit this relationship (Figure 5E). These findings suggest a close relationship between ferroptosis score, neurodegeneration and the phagocyte system.

Following the same method as described above, we categorized the 174 tissue blocks in the spatial transcriptomics data into a high‐ferroptosis score group and a low‐ferroptosis score group. Subsequently, we conducted differential expression analysis and functional enrichment analysis on these two groups. The findings aligned with the cortical snRNA‐seq data, as the brain tissues in the high‐ferroptosis score group exhibited significant enrichment of numerous neurodegenerative disease pathways, including AD, ALS, HD and prion disease (Figures S5A,B and S9). Furthermore, the GO analysis revealed the impact on modules related to the electron transport chain, axo‐dendritic transport, and synapse recycling within the high‐ferroptosis score group (Figure 5F, Table S10). Additionally, we observed a substantial number of upregulated genes in the neurodegenerative disease pathways in the high‐ferroptosis score group (Figure S5C).

### 3.7 Spatial proteomics verifies the ferroptosis score‐neurodegeneration association

Spatial proteomics data^10^ obtained through high‐sensitivity mass spectrometry was utilized to validate our findings at the protein level. To ensure comparability, they conducted spatial proteomics sampling on tissue regions adjacent to the spatial transcriptomics samples. A total of 56 grey matter samples were obtained from 14 progressive MS patients, while nine samples were collected from seven controls. In total, 4541 unique proteins were identified, with 4343 genes overlapping between the spatial transcriptomics and spatial proteomics datasets.

**FIGURE 6:**
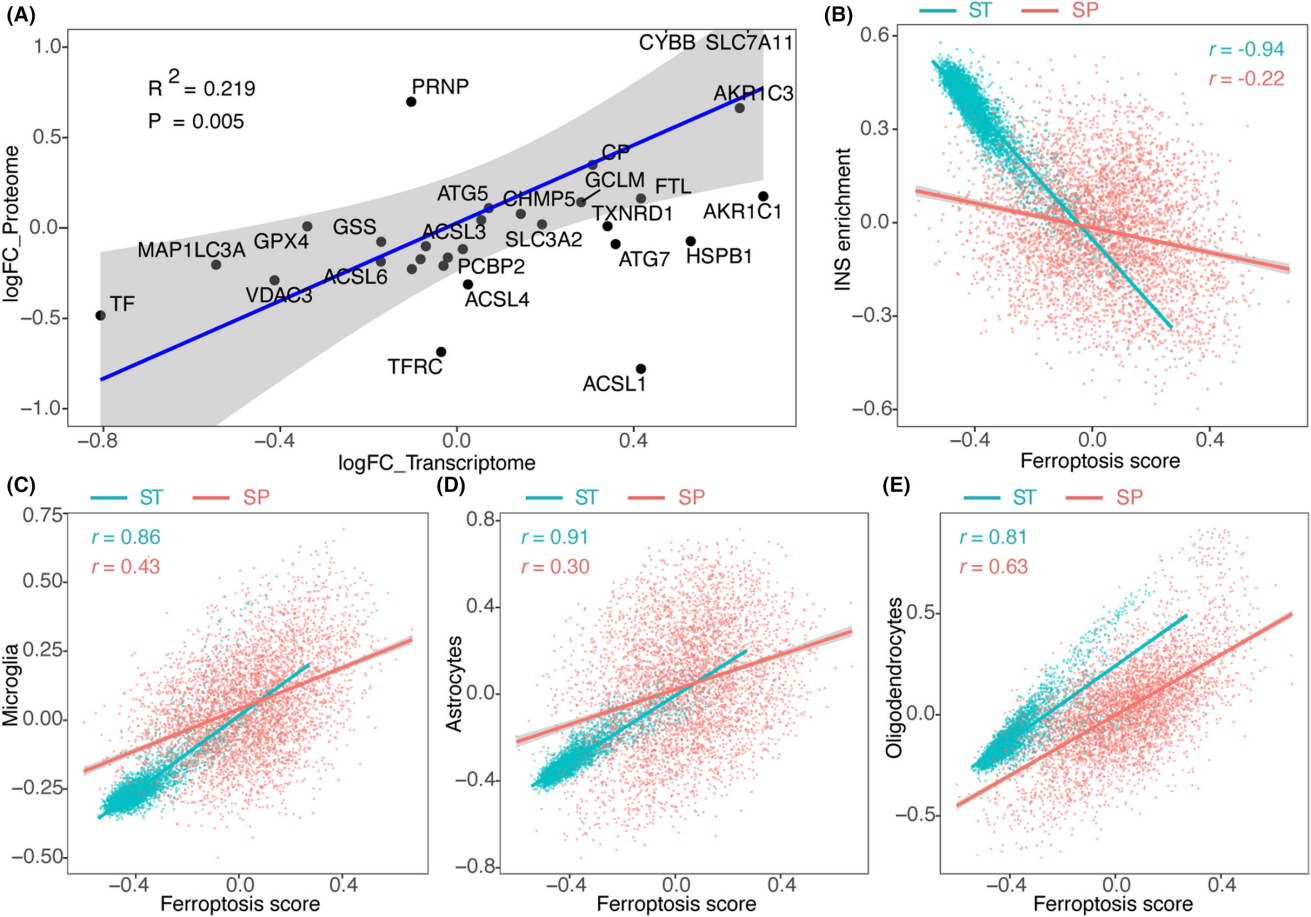
Spatial proteomics verification of the association between ferroptosis score and neurodegeneration. (A) Correlation of log2FC of ferroptosis genes between control and MS in the spatial proteome and the spatial transcriptome. (B) Correlation between ferroptosis scores and intact neuronal signature enrichment in the spatial proteome and the spatial transcriptome. (C–E) Correlation between microglia, oligodendrocytes, and astrocytes with ferroptosis scores in the spatial proteome and the spatial transcriptome. Green dots/line represent the transcriptome; red dots/line represent the proteome. *n* = 4343 jointly detected genes/proteins are plotted. Correlation coefficients were based on Pearson correlations. log2FC, log2FoldChange; SP, spatial proteome; ST, spatial transcriptome.

We conducted a differential expression analysis of spatial proteomics samples from MS patients and controls. Correlation analysis was then performed between the log2FoldChange (log2FC) values of ferroptosis‐related genes in the spatial proteomics and the spatial transcriptomics datasets. The findings revealed a significant positive correlation in the log2FC values of ferroptosis‐related genes between the spatial proteomics and transcriptomics data, providing validation of the transcriptomics data at the protein level (Figure 6A).

Subsequently, we performed the Weighted Gene Co‐expression Network Analysis (WGCNA) to calculate the gene significance (GS) of 4343 genes associated with neurodegeneration (INS enrichment) and ferroptosis score. Spatial proteomics data and spatial transcriptomics data were analysed separately. Consistent patterns emerged at both the transcriptomics and proteomics levels, revealing a negative correlation between ferroptosis score and INS enrichment (Figure 6B). Furthermore, we confirmed a positive correlation between ferroptosis score and microglia, consistently observed in both the transcriptomics and proteomics datasets (Figure 6C). Likewise, a similar directional trend was observed in oligodendrocytes and astrocytes between the transcriptomics and proteomics levels (Figure 6D,E). These findings affirm the reliability of the transcriptomics results through proteomics analysis.

### 3.8 Ferroptosis‐related genes serve as potential markers for MS diagnosis and prognosis

**FIGURE 7:**
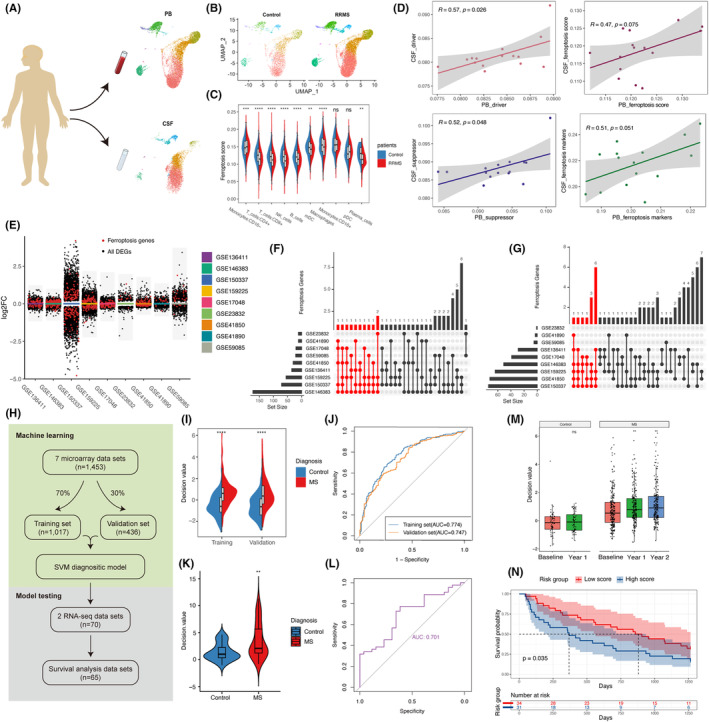
Ferroptosis gene serve as a marker for the diagnosis and prognosis of MS. (A) Schematic diagram of CSF and PB samples (*n* = 130,015 cells from 12 RRMS and 3 Controls). (B) UMAP of cell clusters in control and RRMS. (C) Faceted violin plot comparing ferroptosis scores between RRMS and control in different cell clusters. (D) Correlation of ferroptosis scores and ferroptosis‐related scores in PB with those in CSF of the same individual (*n* = 15). (E) DEGs in 9 bulk RNA datasets of PB. Black dots: all DEGs, red dots: ferroptosis‐related genes. (F) UpSet plot visualizing the properties of intersecting and unique sets of upregulated ferroptosis‐related genes between MS and control, ordered from left to right by amount of evidence. The top 10 upregulated ferroptosis‐related genes with the most evidence are marked in red. The vertical bar chart in the UpSet plot shows the number of ferroptosis‐related genes containing single dataset and co‐occurring datasets, which are indicated by single dots and connected dots in the matrix, respectively. The horizontal bar chart in the UpSet plot shows the total number of ferroptosis‐related genes containing each dataset. (G) UpSet plot visualizing the properties of intersecting and unique sets of downregulated ferroptosis‐related genes between MS and control, ordered from left to right by amount of evidence. The top 10 downregulated ferroptosis‐related genes with the most evidence are marked in red. (H) Machine learning flowchart. (I) Comparison of decision values in training set (*n* = 1017) and validation set (*n* = 436). Control: blue, MS: red. (J) AUC curves of training set and validation set. (K) Comparison of decision values in the test set (*n* = 70). Control: blue, MS: red. (L) The AUC curve of test set. (M) Follow‐up changes of decision values in controls (*n* = 125) and MS paitents (*n* = 690), GSE41850. Compared with the Baseline, the Wilcoxon rank‐sum test was employed. (N) Survival analysis of high‐score group and low‐score group (GSE15245, *n* = 65). *p < 0.05, **p < 0.01, ***p < 0.001. Correlation coefficients were based on Pearson correlations. CSF, cerebrospinal fluid; PB, peripheral blood; RRMS, relapsing–remitting multiple sclerosis; UMAP, Uniform Manifold Approximation and Projection.

To enhance the potential applicability in clinical practice, we examined the ferroptosis scores in both CSF and PB of MS patients. We analysed the available scRNA‐seq dataset^11^ containing paired CSF and PB samples from individuals with relapsing–remitting multiple sclerosis (RRMS) and controls (Figure 7A,B, Figure S6A–C), which enabled the comparison of correlations between PB and CSF.

The analysis revealed that both the CSF and PB ferroptosis scores were lower in MS patients compared to controls (Figure S6D,E). It might indicate a potential redistribution of iron at the overall level in MS patients. Notably, among the CSF and PB samples of MS patients, only classical monocytes (CD16(−) monocytes) exhibited higher ferroptosis scores than controls, while the ferroptosis scores of other cells were lower than those of controls (Figure 7C, Figure S6F,G).

We examined the correlation between the enrichment of ferroptosis‐related genes in the PB and CSF of the same individual. Surprisingly, a significant correlation was observed between PB and CSF in the enrichment of ferroptosis drivers and ferroptosis suppressors. Although statistical significance was not attained, there was a similar correlation trend in the ferroptosis score and ferroptosis markers (Figure 7D). These findings suggest that the state of ferroptosis in PB may reflect the state of ferroptosis in the central nervous system, indicating the potential clinical utility of ferroptosis‐related genes in PB.

We tried to explore the clinical application of ferroptosis‐related genes in the PB using machine learning techniques. To ensure a larger sample size and clinical relevance, we employed bulk RNA data for our machine learning analysis. We conducted a comparative analysis of nine publicly available bulk RNA datasets^12^, ^13^, ^14^, ^15^, ^16^, ^17^, ^18^, ^19^, ^20^ from the Gene Expression Omnibus (GEO) database, comprising both microarray and RNA‐sequencing data, which included MS patients and control subjects. Differential expression analysis was conducted on each data set, comparing MS and control samples, revealing the presence of numerous ferroptosis‐related genes among the DEGs, including both up‐regulated and down‐regulated genes (Figure 7E). The top 10 ferroptosis‐related genes with the strongest evidence were extracted from the sets of up‐regulated and down‐regulated genes, respectively (including all if tied). Subsequently, a total of 24 ferroptosis‐related genes (Table S11) with the strongest evidence were utilized for support vector machine (SVM) learning (Figure 7F,G). All microarray data from 1453 samples were randomly divided, with 70% serving as the training set (*n* = 1017) and 30% as the validation set (*n* = 436) (Figure 7H). By employing machine learning on the 24 ferroptosis‐related genes and diagnostic labels, the model generated decision values as output. Higher decision values indicated a greater likelihood of an MS diagnosis.

The results demonstrated higher decision values for MS in both the training and validation sets compared to the control group, with AUC values exceeding 0.7 for both sets (Figure 7I,J), suggesting that the model has a fair diagnostic performance. Additionally, two completely independent bulk RNA‐sequencing datasets were used for testing, revealing the model's effective diagnostic performance on MS when applied to RNA‐sequencing data (AUC = 0.701) (Figure 7K,L). The model's broad applicability across microarray and RNA‐sequencing data suggests that it primarily relies on distribution differences in the expression of 24 ferroptosis‐related genes, rather than specific expression values. The model's compatibility across detection platforms would significantly enhance its clinical utility.

Moreover, we analysed the evolving trends of decision values in the follow‐up data and observed a gradual increasing trend in the model's calculated decision values over the 1‐year and 2‐year follow‐up period for MS patients (Figure 7M). This shows that from the perspective of the 24 ferroptosis‐related genes, the patients became more and more ‘MS’ during the follow‐up. The decision values of the control group remained relatively stable during the 1‐year follow‐up period (only data for 1 year of follow‐up was available). Additionally, we applied the model to a completely independent dataset^21^ that included precise time‐to‐relapse data for 65 MS patients over 1250 days of follow‐up. We categorized all patients into a high‐score group and a low‐score group based on the mean of their decision values. Our findings revealed that the high‐score group, the more ‘MS’ group, had a higher likelihood of experiencing clinical relapse compared to the low‐score group (*p* = 0.035, Figure 7N). Given the current dearth of effective diagnostic and predictive markers for MS, our results imply that machine learning models utilizing the expression patterns of ferroptosis‐related genes offer a promising avenue for investigation.

## 4 DISCUSSION

Ferroptosis, a type of iron‐dependent programmed cell death discovered in 2012,^38^ is characterized by imbalanced iron homeostasis, metabolic disorder of glutathione, and lipid peroxidation.^38^, ^39^ It has been implicated in the pathogenesis of neurological disorders characterized by iron accumulation, such as AD, PD and ALS.^40^ Notably, significant iron accumulation is observed in MS, including perilesional iron rings on magnetic resonance imaging (MRI) and cortical iron accumulation.^41^ However, the role of ferroptosis in MS patients remains poorly understood. In this study, we elucidate the ferroptosis score in MS patients using snRNA‐seq, spatial transcriptomics and spatial proteomics data. Our findings demonstrate that ferroptosis score exerts relevance on both neuroimmunity and neurodegeneration in MS patients. These results provide a research foundation for ferroptosis‐targeted therapy, offering a promising approach to simultaneously attenuate immune inflammation and neurodegeneration. Additionally, we analysed ferroptosis‐related genes in CSF and PB and developed an MS diagnosis and prognosis model based on 24 ferroptosis‐related genes.

Pathological examination and 7 T MRI imaging revealed that a significant portion of smouldering lesions is surrounded by an iron‐containing rim composed of microglia and macrophages, indicating the potential significance of iron in inflammatory lesion progression.^41^ MS white matter demyelinating lesions are categorized as active (lesion‐wide inflammation), chronic active (inflammation confined to lesion borders), or inactive (lack of inflammatory activity), etc.^42^ We conducted a comparative analysis of the ferroptosis score among different lesion types, including lesion edges, and found that CAE exhibit the highest ferroptosis score compared to other lesion types. Remarkably, this parallels the distribution of iron rings at the edges of active lesions observed on MRI, which implies that active lesions are more susceptible to lesion enlargement.^41^ These findings offer a novel therapeutic strategy by potentially inhibiting lesion development through the use of ferroptosis inhibitors.

We investigate the potential role of ferroptosis score in demyelinating lesions of MS. Myelin, which constitutes the iron‐rich component of brain tissue, is particularly affected in MS. Upon myelin destruction in the brain, phagocytes primarily engulf the iron‐rich myelin fragments.^43^, ^44^ Previous studies have suggested a correlation between iron accumulation in macrophages/microglia and the polarization of the pro‐inflammatory M1 phenotype.^45^ The brain is particularly vulnerable to lipid peroxidation damage.^46^ In the inflammatory demyelination process of the EAE model, rapid reduction in ferritin protein and excessive accumulation of iron in mice trigger lipid peroxidation, resulting in a significant increase in toxic and highly reactive degradation products, such as 4‐hydroxynonenal and malondialdehyde, ultimately leading to ferroptosis of oligodendrocytes and myelin damage.^47^ Studies have also shown that ferroptotic cells secrete factors that enhance T‐cell activation and cytokine production in Th1 and Th17 cells.^6^ The ferroptotic signalling pathway and its resulting phenotypes can be alleviated by anti‐ferroptotic compounds.^6^, ^48^

Our findings indicate that the ferroptosis score are relevant to phagocyte activation and immune inflammation through various pathways. These pathways include T cell activation, interleukin‐1β secretion and enhanced antigen presentation via MHC‐I/II. This interplay establishes a detrimental cycle in which escalating inflammation further exacerbates myelin damage, while the increased production of iron‐containing fragments might intensify ferroptosis. Intervention targeting ferroptosis may offer potential for alleviating or reversing this destructive cycle. Supporting evidence for this therapeutic approach has emerged from animal experiments, wherein deferoxamine (DFO), an iron chelator, demonstrated attenuation of EAE development.^49^ Additionally, reducing the expression of ferroptosis‐related gene *ACSL4* improved behavioural phenotypes and mitigated neuroinflammation in EAE.^6^

Furthermore, we found that ferroptosis also assumes a role in cortical neurodegeneration. Previous evidence has established the significance of ferroptosis in neurodegenerative disorders. However, the precise involvement of ferroptosis in the neurodegeneration of MS remains unclear. The oxidative‐reductive imbalance and oxidative stress induced by ferroptosis may promote cortical neuron degeneration through various mechanisms, including neuronal death, activation of microglial cells, abnormal protein deposition, neuroinflammation, and others.^50^, ^51^, ^52^ In our study, we observed significantly elevated levels of ferroptosis score in neurons of MS patients compared to control cortical neurons. Ferroptosis score in MS cortical neurons is associated with neurodegenerative pathways, potentially affecting the respiratory chain, oxidative phosphorylation, and Aβ clearance. The association between ferroptosis score and MS degeneration was further confirmed through spatial transcriptomics and spatial proteomics analyses. This finding suggests that targeted interventions against ferroptosis might offer the possibility of ameliorating both neuroimmune inflammation and neurodegeneration in MS patients, particularly considering the lack of effective treatments for the latter.

Additionally, for enhanced clinical applicability, we investigated ferroptosis‐related genes in the CSF and PB of MS patients. We identified a significant positive correlation in ferroptosis‐related genes between PB and CSF, suggesting a potential association between peripheral and central. Given the pronounced iron dependency of ferroptosis, alterations in ferroptosis in brain tissue and blood suggest systemic iron distribution abnormalities in patients with MS. Previous studies have partially supported this view, indicating changes in iron levels in both the brain tissue and blood of MS patients.^53^, ^54^, ^55^ We postulate that this connection might be facilitated by monocytes. Notably, monocytes exhibited elevated ferroptosis score in PB compared to the control group, and their ability to communicate between the central and peripheral systems further supports this hypothesis.^43^ To identify effective markers for MS, we developed a diagnostic model based on 24 ferroptosis‐related genes using machine learning techniques. This model demonstrated fair performance in MS diagnosis and relapse prediction. Currently, there is a dearth of reliable diagnostic and prognostic markers for MS (the utility of serum neurofilament light (sNfL) as an MS marker remains limited),^56^, ^57^ and our findings present a novel opportunity to develop a more comprehensive panel of polygenic markers.

## 5 CONCLUSION

Ferroptosis may serve a dual role in both neuroimmune inflammation and neurodegeneration in MS, making it a promising therapeutic target. Additionally, ferroptosis‐related genes in PB have the potential to serve as markers for MS diagnosis and prognosis.

## AUTHOR CONTRIBUTIONS

**Tao Wu:** Formal analysis (lead); investigation (lead); software (lead); writing – original draft (lead); writing – review and editing (lead). **Shangwei Ning:** Methodology (equal); resources (equal); visualization (equal). **Huixue Zhang:** Data curation (equal); validation (equal). **Yuze Cao:** Methodology (equal); validation (equal); writing – review and editing (equal). **Xia Li:** Methodology (equal); software (equal); supervision (equal); writing – review and editing (equal). **Junwei Hao:** Conceptualization (equal); writing – review and editing (equal). **Lihua Wang:** Conceptualization (equal); writing – review and editing (equal).

## FUNDING INFORMATION

This research was supported by the National Natural Science Foundation (82090043 and 81825008 to JH), the National Key Research and Development Program of China (2021YFA1101403), National Natural Science Foundation (81820108014 and 82171396 to LW).

## CONFLICT OF INTEREST STATEMENT

All authors declare no competing interests.

## Supporting information


Figure S1.

Table S1.

## Data Availability

All data in this study are available in Table S1.
