# Supplementary material for: Role of ferroptosis in neuroimmunity and neurodegeneration in multiple sclerosis revealed by multi‐omics data
Source: J Cell Mol Med. 2024 May 27;28(10):e18396. doi: 10.1111/jcmm.18396 (PMC11129625; doi:10.1111/jcmm.18396)
Supplement: Supplementary file 1 — Figure S1. [file JCMM-28-e18396-s001.docx]

**
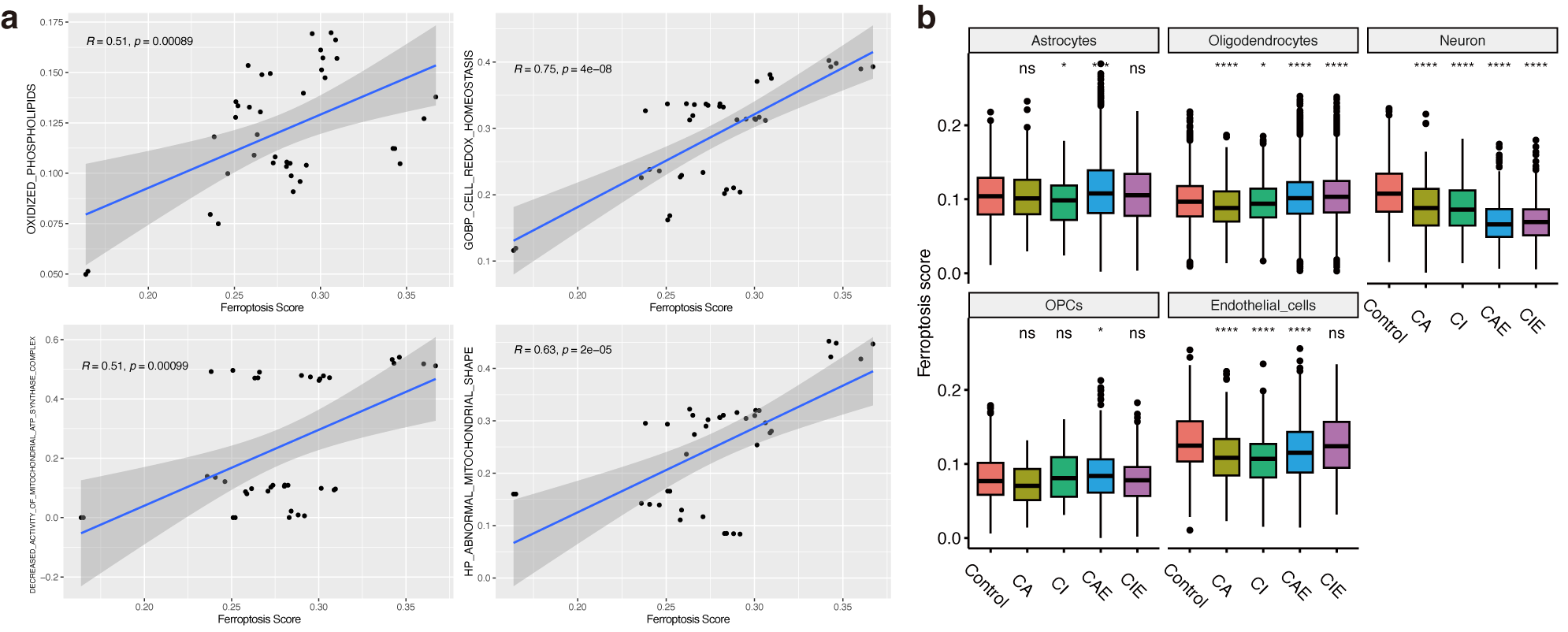
**

**Figure S1 Reliability of ferroptosis scores and the comparison of ferroptosis scores in White matter snRNA-seq data**

**a** Correlation of ferroptosis scores with pathways associated with ferroptosis signatures.. **b** Comparison of ferroptosis scores at different sites in different cell clusters, compared with control (wilcox. test). **p*<0.05, ***p*<0.01, ****p*<0.001. MS, multiple sclerosis; CA, chronic active lesion; CAE, chronic active lesion edge; CI, chronic inactive lesion; CIE, chronic inactive lesion edge.


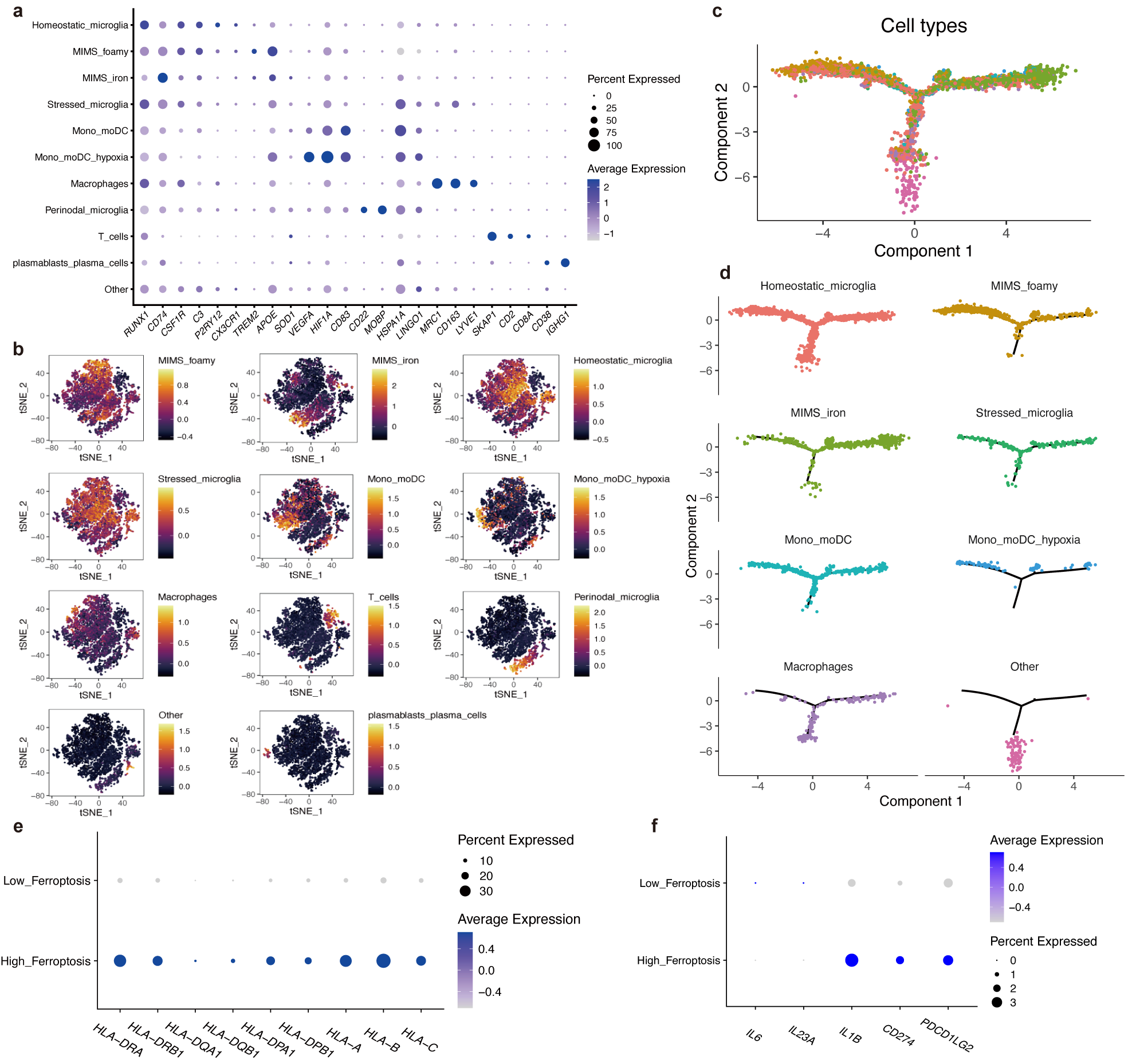


**Figure S2 Analysis of immune cells in white matter snRNA-seq data**

**a** Bubble plot of expression of immune cell markers. **b** Projection of enrichment scores for gene sets of specific immune cell types. **c–d** Projection of cell clusters onto the pseudotime map. **e** Bubble plot of *HLA* gene expression in phagocytes of high-ferroptosis score group and low-ferroptosis score group. **f** Bubble plot of genes of interleukin and PD1 ligand in phagocytes of high-ferroptosis score group and low-ferroptosis score group. *PD-L1*, also known as *CD274*; *PD-L2*, also known as *PDCD1LG2*. mono/moDC, monocytes/dendritic cells; MIMS, microglia inflamed in MS.


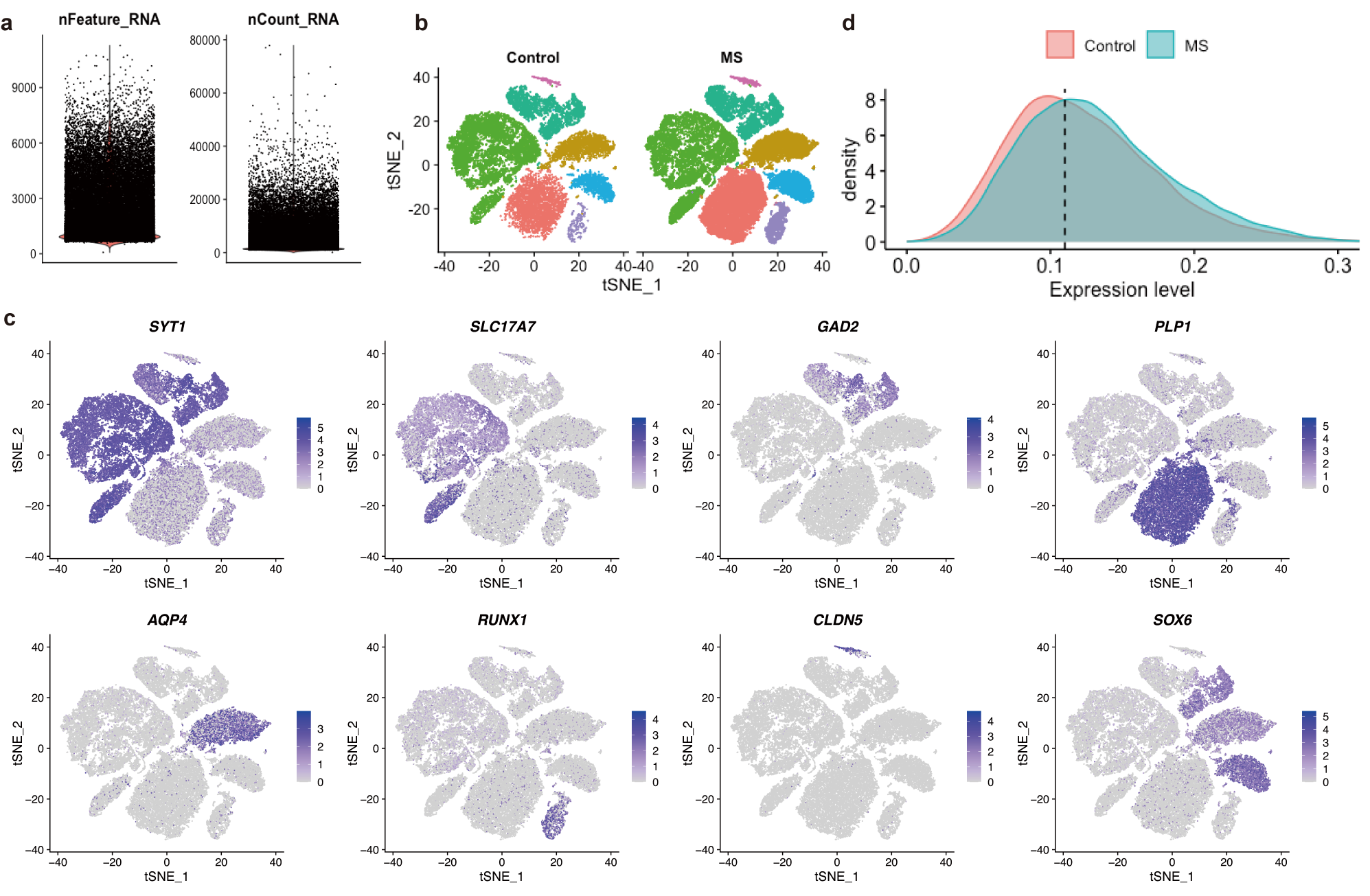


**Figure S3 Quality control of cortical snRNA-seq data**

**a** Features and counts of the cortical snRNA-seq data. **b** tSNE plots of control and MS cell clusters. **c** Projection of specific cell markers. **d** Density plot of ferroptosis scores in control and MS cortex. tSNE, t-distributed Stochastic Neighbor Embedding.


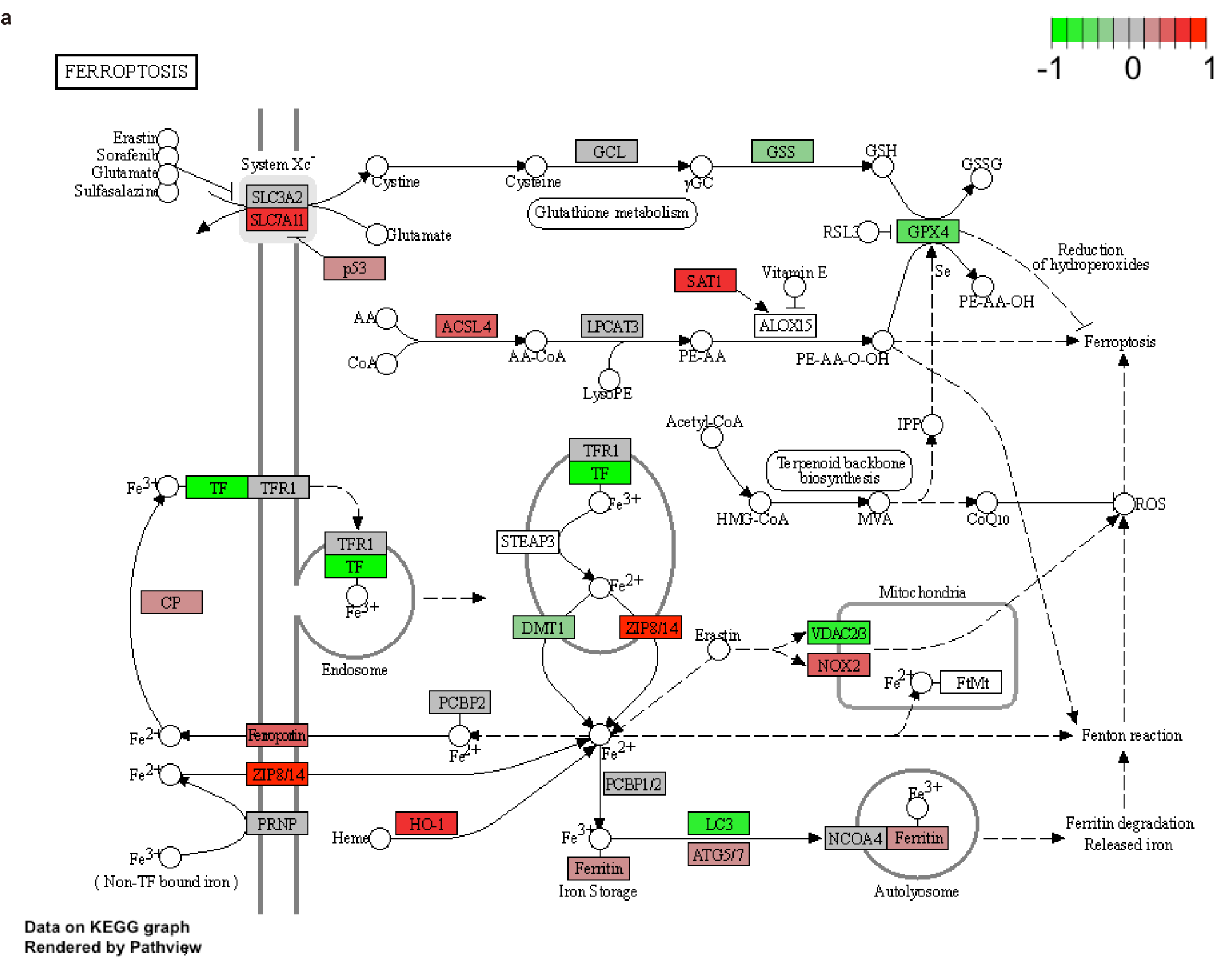


**Figure S4 Changes of ferroptosis-related genes in the MS group compared with the control group in the spatial transcriptomics**

**a** Projection of the changes of ferroptosis-related genes in the MS compared to control onto the KEGG pathway map of ferroptosis.


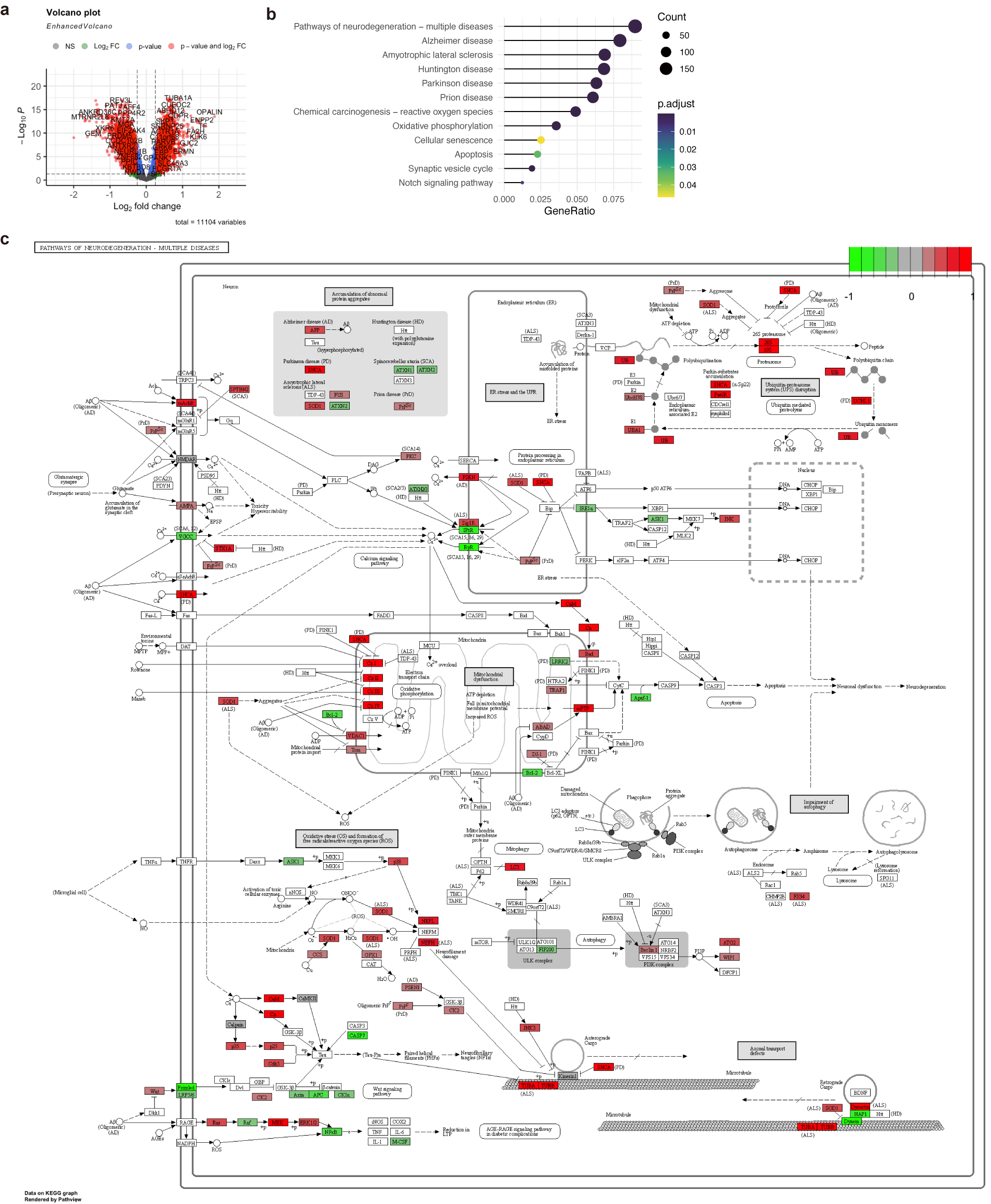


**Figure S5 Comparison of high-ferroptosis score group and low-ferroptosis score group in the spatial transcriptomics**

**a** Volcano plot of DEGs in the high-ferroptosis score group versus the low-ferroptosis score group. **b** KEGG functional enrichment analysis of the high-ferroptosis score group. **c** Projection of the changes of genes in the high-ferroptosis score group compared to the low-ferroptosis score group onto the KEGG pathway map of neurodegeneration. DEGs, differentially expressed genes.


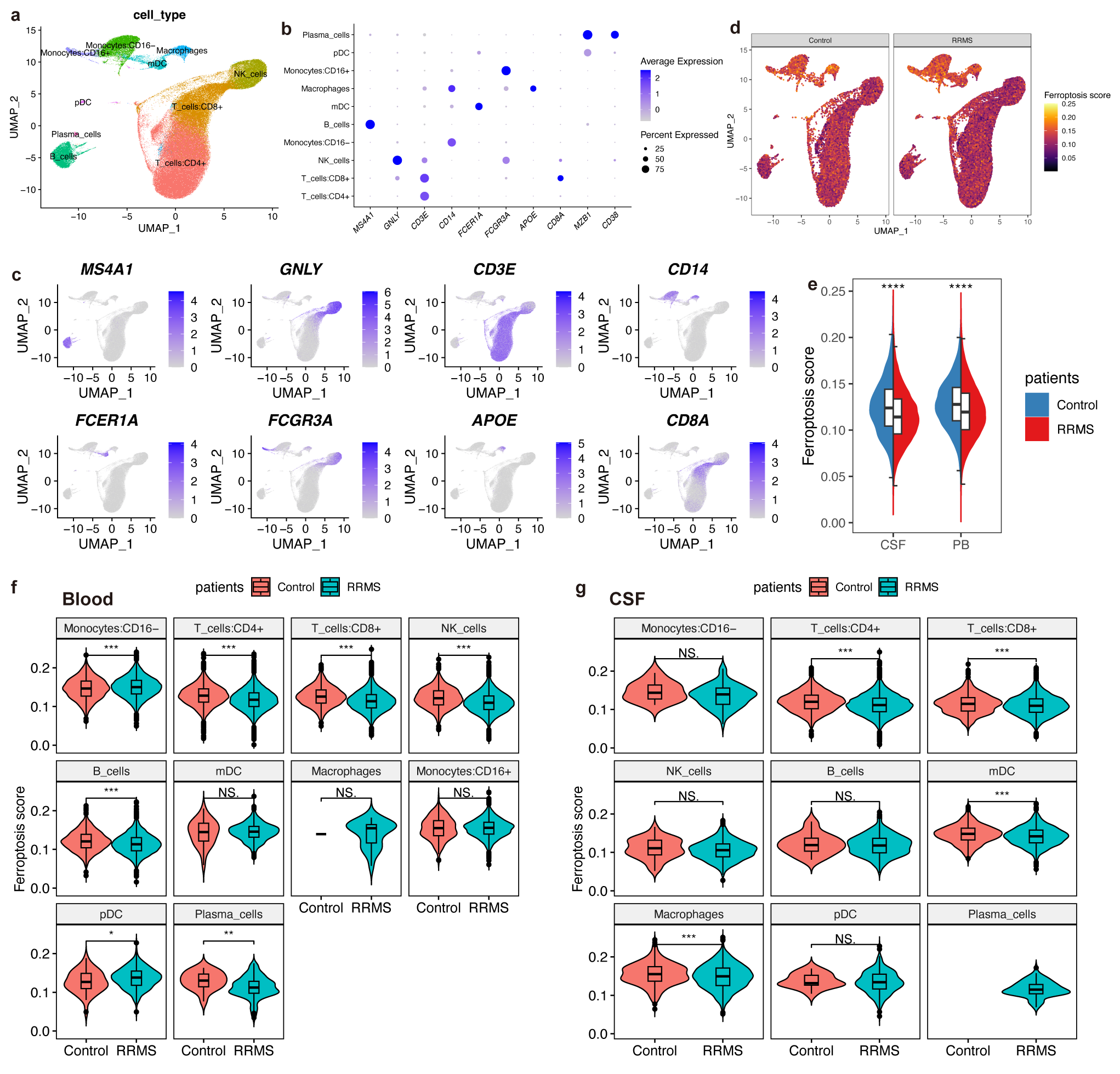


**Figure S6 Changes of ferroptosis scores in cerebrospinal fluid and peripheral blood**

**a** UMAP of cell clusters in CSF and PB. **b** Bubble plot of specific cell markers. **c** Projection of specific cell markers. **d** Projection of ferroptosis scores on UMAP. **e** Comparison of ferroptosis scores between RRMS patients and controls, CSF and PB are shown respectively. **f** Comparison of ferroptosis scores in different cell clusters in PB. **g** Comparison of ferroptosis scores in different cell clusters in CSF. **p*<0.05, ***p*<0.01, ****p*<0.001. RRMS, relapsing-remitting multiple sclerosis; CSF, cerebrospinal fluid; UMAP, Uniform Manifold Approximation and Projection; PB, peripheral blood.
